# Stigma in the health clinic and implications for PrEP access and use by adolescent girls and young women: conflicting perspectives in South Africa

**DOI:** 10.1186/s12889-022-14236-z

**Published:** 2022-10-14

**Authors:** Laura Nyblade, Jacqueline W. Ndirangu, Ilene S. Speizer, Felicia A. Browne, Courtney Peasant Bonner, Alexandra Minnis, Tracy L. Kline, Khatija Ahmed, Brittni N. Howard, Erin N. Cox, Abigail Rinderle, Wendee M. Wechsberg

**Affiliations:** 1 Global Health Division, RTI International, Washington, DC USA; 2 Substance Use, Gender, and Applied Research Program, RTI International, Research Triangle Park, NC USA; 3 Gillings School of Global Public Health, University of North Carolina at Chapel Hill, Chapel Hill, NC USA; 4 Women’s Global Health Imperative, RTI International, Berkeley, CA USA; 5 School of Public Health, University of California, Berkeley, CA USA; 6 Social Statistics Program, RTI International, Research Triangle Park, NC USA; 7 Setshaba Research Centre, Tshwane, South Africa; 8 Department of Psychology, North Carolina State University, Raleigh, NC USA; 9 Psychiatry and Behavioral Sciences, Duke University School of Medicine, Durham, NC USA; 10 Department of Medical Microbiology, Faculty of Health Sciences, University of Pretoria, Pretoria, South Africa

**Keywords:** Stigma, Adolescent girls and young women, Health clinics, HIV prevention, PrEP access, South Africa

## Abstract

**Background:**

Globally, an urgent need exists to expand access to HIV prevention among adolescent girls and young women (AGYW), but the need is particularly acute in sub-Saharan Africa. Oral pre-exposure prophylaxis (PrEP) offers an effective HIV prevention method. In many countries, however, accessing PrEP necessitates that AGYW visit their local health clinic, where they may face access challenges. Some countries have implemented youth-friendly services to reduce certain challenges in local health clinics, but barriers to access persist, including clinic stigma. However, evidence of clinic stigma toward AGYW, particularly with respect to PrEP service delivery, is still limited. This mixed methods study explores stigma toward AGYW seeking clinic services, in particular PrEP, from the perspective of both clinic staff (clinical and nonclinical) and AGYW who seek services at clinic sites in Tshwane province, South Africa.

**Methods:**

Six focus group discussions were conducted with AGYW (43 total participants) and four with clinic staff (42 total participants) and triangulated with survey data with AGYW (n = 449) and clinic staff (n = 130). Thematic analysis was applied to the qualitative data and descriptive statistics were conducted with the survey data.

**Results:**

Four common themes emerged across the qualitative and quantitative data and with both AGYW and clinic staff, although with varying degrees of resonance between these two groups. These themes included (1) clinic manifestations of stigma toward AGYW, (2) concerns about providing PrEP services for AGYW, (3) healthcare providers’ identity as mothers, and (4) privacy and breaches of confidentiality. An additional theme identified mainly in the AGYW data pertained to stigma and access to healthcare.

**Conclusion:**

Evidence is needed to inform strategies for addressing clinic stigma toward AGYW, with the goal of removing barriers to PrEP services for this group. While awareness has increased and progress has been achieved around the provision of comprehensive, youth-friendly sexual and reproductive health services, these programs need to be adapted for the specific concerns of young people seeking PrEP services. Our findings point to the four key areas noted above where programs seeking to address stigma toward AGYW in clinics can tailor their programming.

Globally, an urgent need exists to expand access to HIV prevention among adolescent girls and young women (AGYW), but the need is particularly acute in sub-Saharan Africa where an estimated 4200 AGYW aged 15 to 24 years old acquired HIV every week in 2020 [1]. Within this region, South Africa had the highest HIV prevalence for AGYW (10.4%) [1]. In response to the persistent disproportionate burden of HIV among AGYW, South Africa’s National Strategic Plan for HIV, TB and STIs 2017─2022 prioritizes HIV prevention for AGYW [2]. Oral pre-exposure prophylaxis (PrEP) has been added as an evidence-based tool to the HIV prevention toolbox for AGYW in South Africa and globally [3].

PrEP offers an effective HIV prevention method and unlike condoms does not need to be discussed or negotiated with a sexual partner [4, 5]. In South Africa, however, accessing PrEP can require a prescription, which necessitates young women visiting their local health clinic. Consequently, AGYW who live in economically underserved communities must overcome myriad challenges when seeking PrEP and other sexual and reproductive healthcare (SRH) services. While South Africa has implemented youth-friendly services—such as nurses certified in youth health needs—to reduce some of the challenges in local health clinics, barriers to access persist.

A key barrier to AGYW SRH service access is stigma [6–9]. Stigma is a social process rooted in power that includes labeling, attributing negative stereotypes to people or groups who have been labeled, and othering, which culminates in discrimination[10]. Societal stigma toward AGYW accessing SRH services is often rooted in conservative beliefs about female sexuality, virginity, and purity and on an imbalance of power in sexual and social relationships. For example, sexually active AGYW are often considered to be “bad girls” or “spoiled” [11, 12]. These labels have detrimental effects on AGYW and their families, resulting in gossip, shunning, and the loss of social networks and status [11, 13, 14]. Additionally, societal stigma for sexually active AGYW does not stop at the clinic door, as both clinical and nonclinical staff are also members of their communities and they may unconsciously or consciously reflect societal stigma in the delivery of healthcare to AGYW [15].

Clinic stigma, where AGYW are treated differently than other clients, can manifest across the clinic, emanating from both clinical and nonclinical staff—such as outreach staff, receptionists, or guards—reprimanding young people who come to the clinic for SRH services, gossiping about them, or making them wait longer, among other behaviors [9, 16–22]. Further, provider stigma may result in provision of some services to young people and refusal of other services; this is termed “provider bias” in the family planning literature [9]. Additionally, clinic stigma may extend to AGYW seeking PrEP, given it is an HIV prevention method and therefore associated with sexual activity. A mixed methods study in Tanzania exploring healthcare providers’ willingness to prescribe PrEP to AGYW noted the influence of negative attitudes about adolescent sexuality and a belief that PrEP provision will lead to increased sexual activity [23, 24]. Other studies in East and Southern Africa document that clinic staff continue to hold negative opinions about AGYW’s sexuality and a belief that AGYW cannot properly adhere to SRH routines such as those required for birth control and PrEP [13, 25]. Some providers have expressed that, given the option, they would withhold PrEP access from AGYW altogether, in part to discourage sexual activity [25, 26]. Moreover, because family and community may assume PrEP medicines are for HIV treatment, PrEP users may also face HIV stigma [24, 27, 28].

Although identified as a critical barrier to SRH service access for AGYW, evidence of clinic stigma toward AGYW, particularly with respect to PrEP service delivery, is still limited [24, 29, 30]. Consequently, research is needed to inform strategies for addressing clinic stigma toward AGYW, with the goal of removing barriers to SRH services, including PrEP services. This mixed methods study explores stigma toward AGYW seeking SRH services, in particular PrEP, from the perspectives of both clinical and nonclinical staff and AGYW who seek SRH services at clinic sites in Tshwane province, South Africa.

## Methods

This study used data collected during both the formative (qualitative) and experimental (baseline surveys) phases of the PrEPARE Pretoria Project, a community randomized trial evaluating the efficacy of a multilevel intervention to engage AGYW in PrEP and SRH services. Details of the study have been published elsewhere [7]. The data used in this analysis were collected from 2018 to 2020.

### Formative (qualitative) data

Six focus group discussions (FGDs) were conducted with a convenience sample of AGYW aged 18 to 24 who spoke English, had engaged in condomless sex with a male partner in the past 3 months, were not currently pregnant, were not living with HIV, and who had sought SRH services in Tshwane province. Participants were recruited through community outreach in a cross-section of economically disadvantaged communities in Tshwane where the randomized trial would take place. (FGD participants were excluded from the next phase of the study). In total, 55 respondents were screened for eligibility using a brief field screener and a total of 43, with 5 to 8 participants per FGD, participated. Eligible, interested AGYW were invited to the next FGD that was being held. The FGDs were conducted in English in private settings by trained and experienced facilitators using a semistructured guide. The groups were facilitated by one of two US staff—the study’s Principal Investigator (PI), a White woman; or Co-Investigator (Co-I), a Black woman. The PI has worked in this study area since 2001—leading several projects—and the Co-I has supported projects in this study area since 2008. The aim of these FGDs was to adapt and refine an evidence-based intervention. To ensure this systematic adaptation, the PI (the intervention developer) or Co-I (who has worked closely with the PI on several adaptations) led the FGDs. Each FGD had one primary notetaker—either the aforementioned Co-I or another US Co-I (both Black women) and at least one South African staff member (three Black women who live in the study region) to translate questions or responses in case participants wanted to speak about certain topics or terms in Setswana or Sesotho, which are also common languages spoken in the study area. Topics discussed included PrEP knowledge, access to healthcare services, and stigma while seeking clinic services. After each group, the facilitator, notetaker, and other staff debriefed by reviewing their notes and what was discussed—noting the areas in which saturation had been achieved.

Four FGDs were conducted with a total of 42 clinic staff from two local (city) clinics and two provincial clinics, one FGD per clinic. These mixed groups of convenience-sampled clinical and nonclinical staff comprised administrative clerks (n = 7), a community health worker (n = 1), HIV counselors (n = 5), pharmacists (n = 3), nurses (n = 24), a family physician (n = 1), and a facility manager (n = 1). Staff were included if they were in a position likely to interact with AGYW, interested in participating, and had their manager’s approval to take time away from clinic duties to participate. Final participation was determined by the clinic manager. The mixed groups of clinical and non-clinical staff did not hinder engagement in the discussion by non-clinical staff, who were in many cases the FGD participants with the longest tenure at the clinic. Topics explored included stigma and discrimination in clinics toward AGYW seeking SRH services, including HIV treatment; perceptions of barriers in reaching AGYW, including service delivery and barriers to providing birth control services; and PrEP knowledge, prescription, and dispensing.

The FGDs were audio-recorded and transcribed. Dedoose software (v.8.0.42) was used to manage, code, and analyze of the data. An initial codebook was developed through a combined deductive (based on FGD guides) and inductive (based on the transcripts) process. Intercoder reliability tests were then set up for the two analysts using the test function in Dedoose, with final Kappa scores of 0.72 (clinic) and 0.83 (AGYW). The two analysts then both coded all the transcripts and met to review and compare codes, discuss discrepancies, and agree on a final set of codes for each transcript. Coded data were summarized in visual matrices to identify themes within and across the FGDs.

### Quantitative data

To triangulate the FGD data, we examined baseline quantitative data from the AGYW and clinic staff from the first 6 clinic catchment areas participating in the trial phase of the study.

Baseline surveys were conducted with AGYW (n = 449) aged 16 to 24 who had engaged in condomless sex with a male partner in the past 3 months, were not currently pregnant, were not living with HIV, were interested in PrEP, and had not participated in the formative phase of the trial. For the 16- to 17-year-old respondents, both their assent and consent from their mother or a trusted adult woman at least 25 years old who could serve in loco parentis (“in place of a parent”) was sought. The in loco parentis process enables the young woman to select a female adult (either identified by the young woman herself or by the study staff) to provide consent on her behalf if they are uncomfortable having their mother consent for them. This approach has been used successfully in previous studies in South Africa with adolescents[31–33]. After providing consent or assent, participants completed a baseline survey on a computer tablet via audio computer-assisted self-interviewing (ACASI) in either English or Setswana. The descriptive baseline survey data from the stigma measures collected were shared to triangulate key themes from the FGDs. Stigma measures collected include experienced clinic stigma (ever, past 3 months) and anticipated stigma (ever, past 3 months).

Baseline surveys were conducted with clinical staff (e.g., physicians, nurses; n = 61) and nonclinical staff (e.g., receptionists, clerks; n = 69) who were available at the clinic at the time of the survey (n = 130). The survey assessed SRH knowledge and service provision and attitudes toward PrEP and AGYW seeking PrEP and asked about observations of stigmatizing and discriminatory behavior in their clinic. The survey was self-administered by paper-and-pencil. Nonclinical staff surveys were translated into Setswana for easier comprehension.

### Ethics

The formative phase of the study was approved by the ethics review committees of the South African Medical Association Research Ethics Committee (SAMAREC) and the Office of Human Research Protection at RTI International. The experimental phase of the study was approved by SAMAREC, which served as the Institutional Review Board (IRB) of Record for the intervention, and by the Tshwane District Health Research Committee and the Skills Development for Tshwane Municipal Clinics. All participants provided written informed consent (or assent, if aged 16 or 17) prior to data collection.

## Results

Four common themes emerged across both the qualitative and quantitative data and with both AGYW and clinic staff, although with varying degrees of resonance between these two groups. These themes included (1) clinic manifestations of stigma toward AGYW, (2) concerns about providing PrEP services for AGYW, (3) healthcare providers’ identity as mothers, and (4) privacy and breaches of confidentiality. An additional theme identified mainly in the AGYW data pertained to stigma and access to healthcare.

### Clinic manifestations of stigma toward AGYW

Stigmatizing interactions with clinic staff were commonly described by AGYW in all the FGDs and characterized as rude and harsh, sometimes including shouting.

*They* [nurses] *are very harsh…, most of the time they are so harsh to youth. Where I come from, they are harsh. You can’t even ask for assistance. I don’t think the clinics are a good place to go.*[AGYW, FGD #4]

*Sometimes at the clinic you don’t find the help which you need cause at the clinics you find that when you go there and ask for help, sometimes they just shout at you.* [AGYW, FGD #1]

AGYW also spoke about being subjected to judgmental lecturing—for example, *“they* [clinic staff] *are very judgmental*”—combined with having to respond to what they often perceived as medically unnecessary and excessively intrusive questioning to access services; although one AGYW FGD participant noted that sometimes questions are medically necessary.

*And it’s not their right to say no you cannot have this pill. This pill is for free, whether you had sex 5 times or many times is your own information…they don’t have to force you to say I need this pill because 1,2,3,4, and 5…. The only thing that they need to do is give you what you’re asking for and then explain to you 1,2,3, you’re to take this at this time and what and what.*[AGYW, FGD #3]

Another participant shared:

*First they would ask her about age, um, they get to ask her about confidential, when was the last time you had sex, were you trading money for sex, things like that. They will need the whole information ‘cause they can’t just say “OK, I have that pill, let’s just give it to you”; there are procedures that they have to follow to understand fully about her condition.* [AGYW, FGD #3]

While the prevalence of stigmatizing experiences was a consistent theme expressed across all the AGYW FGDs, it was less commonly discussed in the clinic staff FGDs. When it did appear, it was attributed to “outlier” individual staff, as opposed to a pervasive occurrence or part of the culture of service delivery to AGYW.

*It’s very individual-based on the healthcare worker. We’ve had incidences of healthcare workers who were very judgmental. They would bring Christianity into the picture and make it hard for the adolescent to access, especially younger adolescents.* [Clinic staff, FGD #1]

Or, stigma was discussed as not actually occurring in practice, but rather being anticipated or imagined by AGYW clients. Even though staff might be “silently” judging AGYW, they were not, in their opinion, outwardly expressing it:

*What I’ve realized with my side, I’ve realized they feel as if we are judging them, that’s what I’ve realized. And we…don’t even judge none of them…you know when they come to you their attitude you feel…like they are already ready for the fight even if you are not going to give them an attitude. So that’s my observation. The only thing I can say you know I sometimes you know you feel for them, like in my mind I might be thinking, I wish you could’ve taken a different route, but that will be in my mind and I won’t use it on her, it’s her decision, it’s her choice, we cannot even force anyone to live their life the way we want them to live it.* [Clinic staff, FGD #3]

AGYW survey data confirmed the types of interactions described by AGYW in the FGDs, with 40.5% of AGYW survey respondents indicating they had ever experienced at least 1 of 7 manifestations of stigma (Table 1). Forms of stigma experienced by AGYW respondents ranged from gossip (7.4%) to having had harsh things said because they asked for birth control (20.3%). Also, these were recently occurring experiences; 25.8% of AGYW reported experiencing 1 of 7 manifestations of stigma in the past 3 months (Table 1). Additionally, 12.5% of AGYW respondents reported they had ever been refused services (i.e., not given services by clinic staff [data not shown]).

**Table 1:** Adolescent girls’ and young women’s (AGYW) experiences of stigma at clinics, by lifetime and past 3 months (N = 449)

	Lifetime (Ever)	Past 3 Months
**Experience**	**%**	**%**
The clinic staff said harsh things because I asked for birth control.	20.3	10.0
The clinic staff said harsh things because I asked for an HIV test.	12.0	7.4
The clinic staff treated me badly because of my age.	22.3	14
The clinic staff gossiped about me.	7.4	4.5
The clinic staff looked down on me because of how I looked.	9.1	6.9
The clinic staff looked down on me because of the community that I live in.	7.6	5.4
Felt judged or shamed by clinic staff.	20.3	11.8
**Experienced at least one of the above forms of stigma**	**40.5**	**25.8**

While not readily acknowledged in the clinic staff FGDs, survey data from clinic staff confirmed the presence of stigma in the clinics, specifically for 16- to 17-year-old AGYW seeking SRH services (Table 2), with 39.3% of clinical staff and 56.5% of nonclinical staff reporting they had observed, in the past 3 months, staff unwilling to provide care for 16- to 17-year-old AGYW seeking birth control, sexually transmitted infections (STIs), and antenatal or other care. Also, 42.6% of clinical and 62.3% of nonclinical staff reported hearing staff talking badly about 16- to 17-year-old AGYW seeking these same types of care. Further, roughly a fifth of clinical staff (19.7%) and nearly 50% of nonclinical staff indicated a belief that AGYW deserved to be treated negatively when seeking certain types of services by agreeing with the statement that “talking harshly to AGYW wanting birth control/family planning is right because they are engaging in sexual behavior,” with similar proportions agreeing to a similar statement about AGYW seeking PrEP (data not shown).

**Table 2:** Clinic staff reports of observed stigma toward AGYW aged 16 to 17 seeking health services in the past 3 months, by form of stigma and type of staff

	Form of Observed Stigma			
	**Clinic Staff Unwilling to Care**		**Clinic Staff Talking Badly**	
**Type of Sexual and Reproductive Health Services**	**Clinical Staff (*n*** **= 61)** **%**	**Nonclinical Staff** **(*n*** **= 69)** **%**	**Clinical Staff (*n*** **= 61)** **%**	**Nonclinical staff** **(*n*** **= 69)** **%**
Any type of care	21.3	49.3	19.7	37.7
Antenatal care	24.6	42.0	21.3	43.5
Birth control	29.5	43.5	26.2	49.3
Sexually transmitted infections	24.6	39.1	29.5	46.4
**Observed stigma for at least one of the above types of care**	39.3	56.5	42.6	62.3

### Concerns about providing PrEP services for AGYW

When asked specifically about PrEP for AGYW, clinic staff reflected the same stigmatizing attitudes and stereotype beliefs related to AGYW seeking other SRH services. Clinic staff were concerned that providing PrEP would encourage AGYW to become more sexually active and discourage the use of condoms because they no longer “feared” HIV, which would lead to more pregnancies and STIs

*I don’t know, maybe I’m still backwards. I don’t know why I would allow myself for her to get the PrEP. Maybe I’m not ready to accept the reality that she would be active* [sexually], *you know? *

*It’s like promoting the girls to do whatever they want, which is going to reflect that to them.*

*Another thing that I’m thinking about the PrEP, yes, it would be good to prescribe it, but I’m just worried about these young girls, maybe it would encourage them to be promiscuous.* [Clinic Staff, FGD #3]

*It will increase adolescent pregnancies because they will just not use condoms, because they know they are protected from HIV. They fear HIV more than pregnancy…. Because we see 19-year-olds who come here with a third pregnancy. And that tells us that they are not scared of pregnancy. They can fall pregnant, have these babies, get the social grants, and then they won’t have a problem. But once you say HIV, then it’s a problem for them.* [Clinic staff, FGD #1]

Survey data underscored this concern (Table 3), with nearly 75% of both clinical and nonclinical staff expressing worry that provision of PrEP would lead AGYW to take more sexual risks, and that pregnancy and STI rates would increase. A fifth of clinical staff and two-thirds of nonclinical staff agreed that “it is important to strongly advise AGYW who want PrEP to stop having sex.” Perhaps reflecting these concerns, just over a fifth (21.3%) of clinical healthcare providers said that if they had a choice, they would prefer not to provide SRH services to sexually active AGYW aged 16 to 17, while 14.8% stated the same for unmarried sexually active young women aged 18 to 24.

**Table 3:** Concern about PrEP provision and preferences about provision of sexual and reproductive health services to adolescent girls and young women, by type of clinic staff

Percentage with at least some worry or agreement	Clinical Staff (*n* = 61) %	Nonclinical staff (*n* = 69) %
***If I provide/or if PrEP is provided to adolescent girls (aged 16 to 17) I am worried that…***		
Having access to PrEP will lead them to be reckless or take more sexual risks	73.8	78.3
Pregnancy rates among them will go up because they will stop using condoms if they are using PrEP	75.4	84.1
Other sexually transmitted infections will increase because they will stop using condoms	75.4	82.6
***If*** ***I had a choice, I would prefer not to provide sexual and reproductive health services to…***		
Sexually active adolescent girls aged 16 to 17	21.3	N/A
Unmarried sexually active young women aged 18 to 24	14.8	N/A

### Health providers as mothers

Several of the clinic staff FGDs raised the challenges of providing SRH services to AGYW as they remind them of their own daughters. This implies that being harsh or lecturing AGYW who are seeking SRH services is natural and to be expected because that is how they would treat their own daughters if they sought SRH services.

*I think as mothers, we tend to…personalize, to take it personal. You take this child as your own child…before I attended the AYFS* [adolescent youth friendly service] *course, I was, I was having this thing of, being more of a mother more than a professional, and that is, I think that is the thing that is making the adolescents to stay away from clinics. Because they don’t want to be judged.*[Clinic staff, FGD #2]

*Yeah I think it’s true what you said, nee? We are treating them as our own children. For example, like when a teenager comes in for an abortion, we don’t just write the letter and let her then go, no, we sit down with the child, we counsel her, we counsel her until she changes her mind not to do the abortion.* [Clinic staff, FGD#2]

One clinic staff did note the importance of being cognizant of this potential dynamic and the effect it could have on an AGYW client:

*So we don’t want to treat them as like we are their mothers, because once they see us as parents, then it’s a problem. So, what we usually say is, “Just explain to me ‘cause I want to make sure you fully understand, and I want to make sure everything’s going to work out well. So, I’m here for you, I’m on your side, so tell me, and be honest, I’m not gonna judge you.” So, if you don’t say that, they close up.* [Clinic staff, FGD #1]

AGYW noted the awkwardness they felt in seeking SRH services because : “*most nurses are very old. So, it’s kind of weird, you go to the clinic and consult someone who is the same age with your mother. So, it’s like asking your mother*” [AGYW, FGD #6]. Another AGYW participant perceptively noted that “*I sometimes think that they are trying to be parent figures. Like they’re trying to prevent us from having sex, to abstain ‘cause they think we’re too young, but they’re doing it their own way cause they are being too harsh to us.*” [AGYW, FGD #5]

Survey data from clinic staff underscore that AGYW clients may often be treated as “daughters.” Over two-thirds (88.5%) of clinical staff agreed with the statement, “I would treat the adolescent girl and young woman like my daughter if she were wanting sexual and reproductive health services” (data not shown).

### Lack of clinic privacy and confidentiality

AGYW were also discouraged to use the clinics by concerns around confidentiality and anticipated breaches of confidentiality. The roots of AGYW confidentiality concerns were twofold. The first and most frequently discussed concern related to the physical layout of the clinic and how services were organized/delivered, which led to a lack of confidentiality and unwanted disclosure that “outs” AGYW, making them vulnerable to stigma from other clients and leading to their personal “business” being known by others in the community.

*There was this other girl who came, and I think it was her date to come for the pills and…all of us we know that like there is a certain room, it’s for a certain people, the people who have HIV. So like she* [clinic staff] *just said to her like “you know where you’re supposed to go, you’re going to Room 6 so go there. And stop like bothering me.” So I mean like in front of everyone like everyone was there, they could hear what that person was saying.”*[AGYW, FGD #1]

*Like at reception they ask you “why are you here to do?” when you say “I’m sick” they say “be specific, gonna have be specific” you say “I’m here for that pill” and people behind my back they are listening, people like my neighbors, my friends, my street mates, the whole place is gonna know that girl is on protected pills, she’s having sex…here and there* [multiple sexual partners]*…not knowing that I can be with my partner, trusting my partner, to find out that my partner is doing things, so I’m protecting myself from the person that I love…I moved from one clinic because I knew that my neighbors go to that clinic. So, I go where they don’t know me.”* [AGYW, FGD #3]

The second confidentiality concern was the anticipated behavior of clinic staff themselves, in the form of gossip and sharing of information with other staff and beyond the clinic about an AGYW’s clinic visit, including why they needed services. This was a particular concern if clinic staff were neighbors or relatives of the AGYW.

AGYW shared:

P *We’re scared they’re gonna talk*.

P *Yeah, I’ll say no because some nurses are being rude. Yeah, and can discuss your personal issues*.

P *Yeah, they will go around breaking* [gossiping] *about you, or if you have HIV/AIDS or so on and so on and so on*

Moderator* And what kind of people do they tell? Is it other people in the clinic?*

P *Yeah, other people in the clinic or nurses and some nurses come around your place, [where you live] yeah.* [AGYW, FGD #3]

*But mostly in public hospitals and public clinics they don’t take their roles serious or patient/nurse confidentiality and all that. They might find that okay you go in there for help but then they don’t take it as if you’re there for help, they take your information to someone else then someone else, then to someone else and someone else then in no time then you might find out that okay, people already know, already know that you’re at the clinic for help, like most patients, you are there for family planning, they say you must go to, this and that, yeah, it’s a problem a really big problem.* [AGYW, FGD#2]

The anticipated stigma (fear) of clinic staff breaching confidentiality was reported by 23.4% of AGYW as having ever kept them from getting healthcare, while 17.4% reported this fear as keeping them from getting healthcare in the past 3 months (Table 4). However, reports of actual breaches of confidentiality were lower, with 7.4% of AGYW reporting they had ever been gossiped about by clinic staff, and AGYW reporting that clinic staff had told family (4.9%) and other people in the community (4.5%) that they had visited the clinic (data not shown).

**Table 4:** Impact of experienced and anticipated stigma on healthcare utilization by adolescent girls and young women, by lifetime and past 3 months (*n* = 449)

Not able to get health care because…	Lifetime (Ever) %	Past 3 months %
**Anticipated**		
You were scared that the clinic staff would share your private information	23.4	17.4
You were afraid that others in the community would see you	18.5	13.1
You were afraid that people would spread rumors about why you went to the clinic	20.7	16.3
**Reported at least one of the above anticipated stigma items**	34.5	27.0
**Experienced**		
The nurses and clerks were harsh	38.1	24.7
The nurses were not friendly to young women like you	33.4	22.7
Have you ever stopped going for services at a healthcare clinic because you were judged or shamed?	12.3	10.7
**Reported at least one of the above experience stigma items**	47.8	34.5
**Reported experiencing any anticipated or experience stigma**	56.4	40.8

While concerns about confidentiality were repeatedly discussed in the AGYW FGDs, this topic was not a key theme in the clinic staff FGDs. However, both clinical (19.7%) and nonclinical (43.5%) staff reported observing clinic staff disclosing the health or sexual activity status of AGYW clients in the past 3 months. Additionally, over a quarter (27.9%) of the clinical staff were not sure that their own results would be kept private if they took an HIV test in their facility (data not shown).

### Impact of stigma on AGYW service utilization

While clinic staff indirectly acknowledged that “beliefs that we are judgmental” may keep AGYW from seeking services, AGYW were more explicit in describing how anticipated and experienced stigma keeps them away from needed SRH services.

*So, when you go to the clinic most of the time, you get those nurses…she would look at you just like and then, “why do you need this thing? You are too young to be using this thing!” So that’s what most of us, it holds us back. Like, you are too scared to talk to anyone or too scared to go and find help. You will just stay back and stay out of that thing you wanted to do.* [AGYW, FGD#3]

The negative effect on access to healthcare of both anticipated and experienced stigma at health clinics is reflected in the data from AGYW survey respondents (Table 4), where 34.5% report having ever not been able to get healthcare because of one of three forms of anticipated stigma and 47.8% have ever not been able to get healthcare because of experienced stigma. The recency of the anticipated and experienced stigma is underscored by the reported occurrence in the past 3 months of 27% anticipated and 34.5% experienced. When anticipated and experienced stigma are examined together, over half (56.4%) of AGYW reported that at least one of six forms of anticipated or experienced stigma had ever kept them from accessing healthcare, and 40.8% reported this occurring in the past 3 months (data not shown).

## Discussion

The high rates of HIV acquisition among AGYW in South Africa and other countries in sub-Saharan Africa require a continued focus on increasing AGYW’s access to HIV prevention. PrEP offers an important tool to prevent HIV, particularly for AGYW, because it can be used without involving a young woman’s partner. In South Africa, there are programs that offer PrEP outside health clinics in schools and communities[34]. However, for many AGYW, accessing PrEP requires interacting with public health clinics, necessitating they navigate multiple clinic access barriers, including stigma. Understanding the prevalence, nature, and effect of clinic stigma on AGYW is necessary to address barriers to clinic use and design clinic-level stigma-reduction interventions. While there is a growing literature on AGYW clinic stigma, most studies are either qualitative or quantitative in nature and few triangulate the findings across both AGYW and clinic staff [24, 26, 30, 35–41]. Our mixed methods examination adds to the literature by exploring AGYW clinic stigma from the perspective of both AGYW and clinic staff and then triangulating the qualitative and quantitative data. Additionally, it is important to recognize that clinic stigma happens in interactions with both clinical and nonclinical staff; consequently, we included both groups of staff in the study.

Common forms of AGYW clinic stigma described in AGYW FGDs included harsh, rude, and judgmental interactions of a lecturing nature with staff and intrusive questioning perceived as medically unnecessary, which echoes the findings in other studies [8, 23, 42–44]. The AGYW survey data confirmed the presence of stigma, with over a quarter of AGYW reporting having experienced clinic stigma in the past 3 months and 40% reporting having ever experienced clinic stigma. While the clinic staff FGDs described less clinic stigma than the AGYW FGDs, staff survey data painted a different picture, whereby staff reported observing stigma toward 16- to 17-year-olds seeking SRH services in the past 3 months in similar proportions to the experienced stigma reported in the AGYW survey data.

Societal norms surrounding AGYW’s sexuality and sex outside of marriage are at the heart of AGYW clinic stigma, as seen in the data related to stigma around providing SRH services, including PrEP, to AGYW. This pattern has been described elsewhere [12]. Linked to this stigma are perceived stereotypes of sexually active AGYW as being promiscuous and irresponsible and beliefs that provision of SRH services, including family planning and PrEP, would only serve to encourage riskier and more frequent sex, as seen in the clinic staff data and as documented elsewhere [9, 25, 26, 45, 46].

The data also illuminate the challenges that healthcare providers face in navigating their dual roles and identities as medical professionals and community members, as well as being “mothers,” when providing SRH services to AGYW. Healthcare providers may struggle to leave at the clinic door societal norms that shape their personal beliefs and they may feel a sense of responsibility to uphold and encourage the moral character of AGYW clients, as expected by their community. This responsibility may contradict their professional responsibilities to provide AGYW stigma-free SRH health services. The result of navigating this challenge may be provision of the service but with an accompanying undercurrent of stigma. The challenge of navigating dual roles was clearly illustrated in the data through providers’ descriptions of a certain “mother-daughter” dynamic to their relationship with AGYW clients. Scolding and lecturing may be expected of a mother within the bounds of their home, but when the mother-daughter dynamic is brought into the clinic setting it may present a barrier to AGYW accessing services [42, 43, 47]. AGYW noted the mother-daughter relationship and the discomfort and awkwardness in SRH service access when it feels as if they are asking their mother for PrEP or birth control. When SRH providers hyperindividualize their clients by relating to them as daughters, it may facilitate their rationalization for scolding and other stigmatizing behaviors and mentally compartmentalizing those behaviors as not stigmatizing.

A key theme identified from the AGYW data—which is in turn both a form of enacted stigmatizing behavior and a cause of anticipated stigma—was breaches of confidentiality by clinic staff and lack of privacy at clinics that exposed AGYW to stigma from other clients and the community more broadly. This echoes the findings from other studies concerning the importance of provider confidentiality and privacy and its link to stigma, including how this can lead clients in need of services to avoid services, seek them outside the clinic (e.g. with pharmacists or traditional healers), or travel a distance outside of their communities to receive them [48–50]. Multiple studies have demonstrated that adolescents place great value on the trustworthiness of providers to maintain confidentiality and privacy in the healthcare setting, in addition to the negative behaviors and attitudes of the providers [47, 49, 51]. This is further supported by our findings that while AGYW survey reports of breaches of confidentiality were low, even one breach will signal to other AGYW that the clinic is not to be trusted and fuel fears of stigma. Both the physical layout of a clinic and AGYWs’ trust that staff will maintain confidentiality are intertwined with the anticipation and experience of clinic stigma by AGYW. Consequently, these aspects need to be incorporated into clinic stigma-reduction interventions.

This study has several limitations regarding the data. First, convenience sampling was used for both the AGYW and clinic staff FGDs and they were conducted in English as the primary goal of these groups was to adapt and refine an evidence-based intervention. This may have limited the diversity of the AGYW participants and potentially comprehension of the discussion questions. However, in all the FDGs, at least one other staff member was present to translate questions or responses in case participants wanted to speak about certain topics or terms in Setswana or Sesotho, which are also common languages spoken in the study area. Second, as the AGYW were drawn from across Tshwane province and had experience seeking SRH services in the province at similar clinics, their reflections on clinic experiences cannot be assumed to be related to the specific clinics from which staff data were collected. Third, while clinic managers were requested to release a mix of clinical and nonclinical staff for participation in the FGDs, busy clinic schedules and staff conducting outreach services or who were otherwise away from the clinic may not have had a chance to participate. Fourth, the data are cross-sectional; consequently, they capture the situation of stigma in the lives of the AGYW and at the clinics in a moment of time rather than across time. Lastly, social desirability bias, particularly among clinic staff, is always a challenge with data on health facility stigma. Despite these limitations, the strength of this study is having collected mixed methods data across both AGYW and clinic staff, which allows for triangulation of the data.

## Conclusion

Both AGYW and clinic staff report the presence of PrEP stigma. AGYW described more and different manifestations of stigma in the qualitative data compared with clinic staff. However, the survey data shows clinic staff reporting a similar level of observed stigma as AGYW report experiencing. Although awareness has increased and progress has been made around the provision of comprehensive youth-friendly SRH services, these programs need to be adapted for the specific concerns of young people seeking PrEP services, particularly a focus on addressing clinic stigma [51–53]. Although specific clinic interventions targeted at AGYW and PrEP stigma are limited, a solid foundation of evidence-based clinic stigma-reduction interventions exists and provides readily adaptable approaches and tools[54]. For example, the Health Policy Plus (HP+) Total Facility HIV stigma-reduction intervention [55] has a three-phased approach that includes assessment of the drivers of HIV stigma in a specific facility that includes a modular, easily adaptable participatory stigma-reduction curriculum that is delivered by trained staff and clients from a facility and a process for the clinic to analyze, deepen and institutionalize new found understanding of stigma and action developed by staff through the participatory training; for example by reexamining the physical layout and way in which services are delivered. This approach has been adapted in Thailand[56], Ghana (with a focus on sexual and gender diversity stigma)[57], Bangladesh (with a focus on youth SRH stigma)[58] and India[19, 59]. Guided by the findings of our mixed methods study, we readily adapted the participatory training component of the HP + Total Facility Approach [55]to focus on AGYW and PrEP stigma and we are currently testing it a study in South Africa [7].

## Data Availability

Data and materials are available on request from Dr. Wendee Wechsberg at wmw@rti.org.

## Abbreviations

ACASI: audio computer-assisted self-interviewing.
AGYW: adolescent girls and young women.
AYFS: adolescent youth friendly service.
FGD: focus group discussion.
PrEP: pre-exposure prophylaxis.
SAMAREC: South African Medical Association Research Ethics Committee.
SRH: sexual and reproductive health.
